# The association between changes in cerebral hemodynamics and cerebrovascular complications during ECMO treatment in neonates

**DOI:** 10.1371/journal.pone.0314166

**Published:** 2024-12-06

**Authors:** Jian-Feng Liu, Yi-Nan Liu, Ya-Ting Zeng, Zhe-Yuan Gao, Heng Cai, Yi-Rong Zheng, Qi-Liang Zhang, Qiang Chen

**Affiliations:** Department of Cardiac Surgery, Fujian Children’s Hospital (Fujian Branch of Shanghai Children’s Medical Center), College of Clinical Medicine for Obstetrics & Gynecology and Pediatrics, Fujian Medical University, Fuzhou, China; Sapienza University of Rome: Universita degli Studi di Roma La Sapienza, ITALY

## Abstract

**Objective:**

To investigate whether changes in cerebrovascular hemodynamic parameters during extracorporeal membrane oxygenation (ECMO) treatment in neonates are associated with the occurrence of cerebrovascular complications.

**Methods:**

This study selected neonatal patients who received ECMO treatment at a pediatric hospital in China from June 2021 to June 2024. Relevant clinical data were collected from the electronic medical record system. Data from cranial ultrasound examinations before and during ECMO treatment, as well as the occurrence of cerebrovascular complications, were collected for further analysis.

**Results:**

A total of 37 neonates were enrolled in this study. Among them, 15 neonates who developed cerebrovascular complications during ECMO were included in the complication group, while the remaining 22 neonates were included in the non-complication group. The age difference between the two groups was statistically significant. The systolic blood pressure coefficient of variation (SBP-CV) and diastolic blood pressure CV (DBP-CV) were significantly higher in the complication group compared to the non-complication group. Additionally, the anterior cerebral artery peak systolic velocity CV (ACA-PSV-CV) and ACA resistance index CV (ACA-RI-CV) were significantly higher in the complication group than in the non-complication group. However, there was no significant difference in the ACA end-diastolic velocity CV (ACA-EDV-CV) between the two groups. The receiver operating characteristic (ROC) curve analysis of risk factors for cerebrovascular complications indicated that the area under the curve (AUC) for ACA-RI-CV was 0.765 (95% CI: 0.608–0.923, p = 0.007). The AUC for ACA-SBP-CV was 0.815 (95% CI: 0.666–0.964, p = 0.001). Moreover, when ACA-RI-CV and ACA-SBP-CV were combined, the AUC was 0.873 (95% CI: 0.758–0.988, p<0.001).

**Conclusion:**

High ACA-RI-CV and ACA-SBP-CV were associated with the occurrence of cerebrovascular complications during ECMO treatment in neonates. The combined detection of ACA-RI-CV and ACA-SBP-CV had a predictive role in the early identification of cerebrovascular complications in neonatal ECMO patients.

## Introduction

Extracorporeal membrane oxygenation (ECMO) has increasingly become a critical treatment option for neonates with severe, reversible respiratory or circulatory failure that is unresponsive to conventional medical therapies [1,2]. ECMO provides physicians with the ability to offer temporary cardiopulmonary support when conventional therapies are insufficient, thereby improving survival rates in these patients. However, neonates receiving ECMO remain at risk for various complications. Previous studies have reported that the incidence of neurological complications in neonates during ECMO ranges from 10% to 52% [3,4]. The mechanisms underlying neurological complications during ECMO therapy may involve the need for continuous anticoagulation, the instability of non-pulsatile mechanical blood flow, and disruptions in cerebrovascular autoregulation. These factors can contribute to neurological events such as cerebral hemorrhage or ischemia [5,6]. Neonates are at an especially higher risk of neurological complications due to the immaturity of their cerebrovascular system and cerebral blood flow autoregulation mechanisms [7].

With the increasing application of ECMO technology in recent years, early diagnosis and intervention for cerebrovascular complications have become critical for improving treatment outcomes and patient prognosis. Research by Chin et al. indicates that early detection and timely intervention through neurological monitoring during ECMO can effectively prevent disease progression and improve neurological outcomes at the time of discharge [8]. While some studies in adult and older pediatric ECMO populations have shown that changes in cerebrovascular hemodynamics may be linked to cerebrovascular complications, research specifically focusing on neonates remains relatively limited [9,10]. Therefore, our study aims to systematically analyze the potential relationship between changes in cerebrovascular hemodynamic parameters and cerebrovascular complications in neonates during ECMO treatment using quantitative methods.

## Methods

### Study population

This is a retrospective study focused on the neonatal ECMO population. The study was approved by our institution’s ethics review board. Due to its retrospective design, the Ethics Committee waived the requirement for informed consent, as the study posed minimal risk to participants, and all data were analyzed anonymously to ensure confidentiality. Neonates who received ECMO treatment at our hospital from June 2021 to June 2024 were selected as the study population. All treatment decisions during ECMO treatment were made by the same treatment team following a consistent treatment protocol based on the patient’s condition. All neonates eligible for ECMO were treated with veno-arterial ECMO (V-A ECMO), adhering to treatment guidelines established in prior studies [11]. Neonates were included in the study if they met the following criteria: 1. neonate patients; 2. received ECMO treatment at our hospital; 3. underwent cranial ultrasound examination before ECMO treatment and daily for the first three days after ECMO initiation. Exclusion criteria included: 1. neonates with hypoxic-ischemic encephalopathy, intracranial complications (hemorrhage, ischemic injury, hydrocephalus, or conditions requiring neurosurgery) before ECMO treatment; 2. lack of cerebral blood flow velocity-related cranial ultrasound data necessary for the study; 3. incomplete clinical baseline data required for the study.

### Cranial ultrasound examination

All patients were examined using the same bedside ultrasound equipment (Vivid iq, General Electric Company, USA). Each Doppler parameter was averaged over 4 to 5 cardiac cycles. The probe scanned through the anterior fontanelle to measure anterior cerebral artery (ACA) blood flow parameters and assess the presence of intracranial complications bilaterally. Cerebral hemodynamic parameters included peak systolic velocity (PSV), end-diastolic velocity (EDV), and resistance index (RI) of the ACA. The formula for RI is RI = (PSV-EDV)/PSV. Each neonatal cranial ultrasound examination, for those who developed cerebrovascular complications during ECMO treatment, was reviewed consistently by two experienced senior neonatal cranial ultrasound specialists to confirm cerebrovascular complications. According to our neuromonitoring detects strategy during ECMO treatment, all neonates undergoing ECMO received a bedside cranial ultrasound before cannulation and routine bedside cranial ultrasound examination daily for the first three days during ECMO treatment. All cranial ultrasound measurements in this study were performed by the same experienced cranial ultrasound specialist, who was blinded to the study participation status of the neonates.

### Data collection

In this study, relevant clinical data of the included neonates were collected through our electronic medical record system. These data included basic information such as gestational age, gender, age and weight at the time of cannulation, and primary diagnoses. Invasive blood pressure monitoring data were recorded every hour for the first three days after the initiation of ECMO treatment. Monitoring indicators included systolic blood pressure (SBP) and diastolic blood pressure (DBP). Besides basic clinical information, the study also collected data including PSV, EDV, and RI of the ACA. Variability indicators such as the coefficient of variation (CV) for SBP, DBP, and RI were calculated (CV = standard deviation (SD) / mean (μ)). All data collection adhered to ethical and privacy protection principles. A dedicated research assistant was responsible for data collection and organization to ensure data accuracy and security.

### Statistical analysis

Quantitative variables were presented as means and SD, while categorical variables were shown as frequencies and percentages (%). The normality of the variables was assessed by measuring skewness and kurtosis. For variables that followed a normal distribution, an independent sample T-test was used for comparisons. For non-normally distributed data, we selected the Mann-Whitney U test rather than other non-parametric tests because it is well-suited for smaller sample sizes and did not require the data to follow a normal distribution. The chi-square test or Fisher’s exact test was used to compare categorical variables between two groups. Receiver operating characteristic (ROC) curve analysis was performed to evaluate the predictive ability of blood pressure variability and cerebral blood flow velocity variability indicators for cerebrovascular complications in neonates undergoing ECMO treatment. This analysis included calculating the area under the curve (AUC) for each variable and determining the optimal cutoff values to maximize sensitivity and specificity. All data processing and analysis were conducted using IBM SPSS Statistics software version 25, with statistical significance set at p<0.05.

## Results

**Fig 1:**
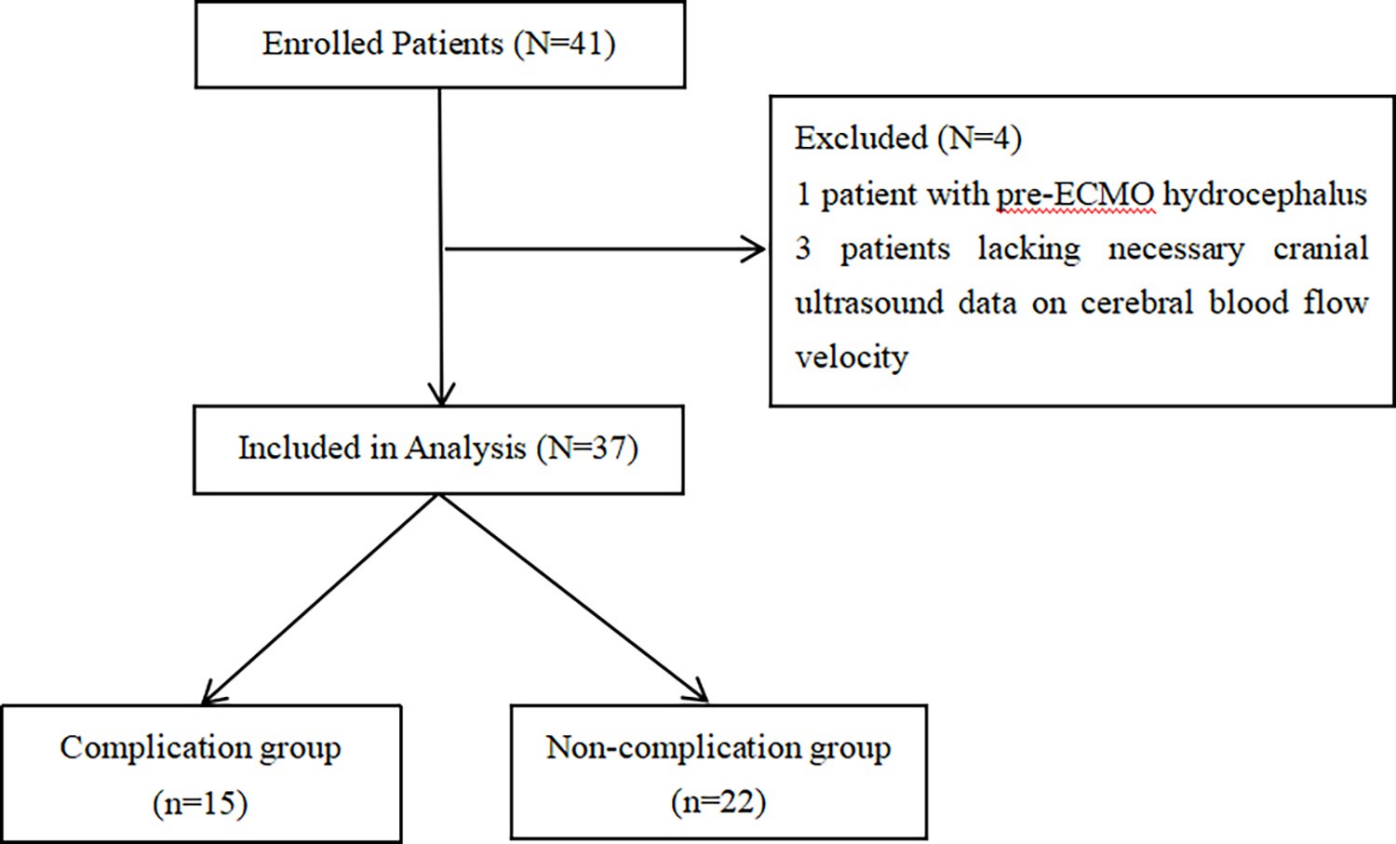
The flowchart shows the selection process of the study participants.

A total of 41 neonates meeting the inclusion criteria were enrolled in this study. Four neonates were excluded from the study: one patient due to pre-ECMO hydrocephalus and three patients because of missing essential cranial ultrasound data. Ultimately, 37 neonates were included in the analysis. Of these, 15 neonates who developed cerebrovascular complications during ECMO treatment were assigned to the complication group, while the remaining 22 neonates were placed in the non-complication group (Fig 1). The age of neonates in the complication group was 2 days (IQR: 1 to 2), and in the non-complication group was 2 days (IQR: 1 to 5); the age difference between the two groups was statistically significant (p<0.05). The weight of neonates in the complication group was 3.1±0.6 kg, and in the non-complication group was 3.3±0.7 kg; the weight difference between the two groups was not statistically significant (p>0.05). Additionally, there were no statistically significant differences between the two groups in terms of gestational age and total ECMO runtime (p>0.05) (Table 1).

**Table 1:** Clinical data of all patience.

	Complication group (n = 15)	Non-complication group (n = 22)	p value
Gestational age, w	37.7±1.8	38.2±1.4	0.332
Male gender, n (%)	10 (66.7)	17 (77.3)	0.708
Weight, kg	3.1±0.6	3.3±0.7	0.384
Premature infant, n (%)	5 (33.3)	4 (18.2)	0.438
Age at cannulation, d	2 (1 to 2)	2 (1 to 5)	0.015
Primary diagnoses, n (%)			
PPHN	5 (33.3)	10 (45.5)	0.733
CHD	3 (20.0)	5 (22.7)	
CDH	2 (13.3)	1 (4.5)	
ARDS	3 (20.0)	2 (9.1)	
Others	2 (13.3)	4 (18.2)	
Total ECMO runtime, h	80.4±38.2	82.9±54.5	0.928

PPHN: Persistent pulmonary hypertension of the newborn; CHD: Congenital heart disease; ARDS: Acute Respiratory Distress Syndrome; CDH: Congenital diaphragmatic hernia.

Table 2 lists the comparisons of the main cerebral blood flow velocity and blood pressure variability data measured during ECMO treatment between the two groups. The ACA-SBP-CV and ACA-DBP-CV were significantly higher in the complication group compared to the non-complication group (p<0.05). The ACA-PSV-CV and ACA-RI-CV were also significantly higher in the complication group than in the non-complication group (p<0.05). However, there was no significant difference in ACA-EDV-CV between the two groups (p>0.05).

**Table 2:** Comparison of clinical data between the two groups.

	Complication group (n = 15)	Non-complication group (n = 22)	p value
SBP-CV (%)	19.3±2.6	15.5±3.5	0.001
DBP-CV (%)	17.9±3.1	14.8±3.3	0.005
ACA-PSV-CV (%)	12.7±6.5	6.7±3.3	0.004
ACA-EDV-CV (%)	14.5±15	8.5±5.1	0.166
ACA-RI-CV (%)	10.5±7.8	6.4±3.4	0.041

SBP: Systolic blood pressure; DBP: Diastolic blood pressure; ACA: Anterior cerebral artery; PSV: Peak systolic velocity; EDV: End-diastolic velocity; RI: Resistance index; CV: Coefficient of variation.

**Fig 2:**
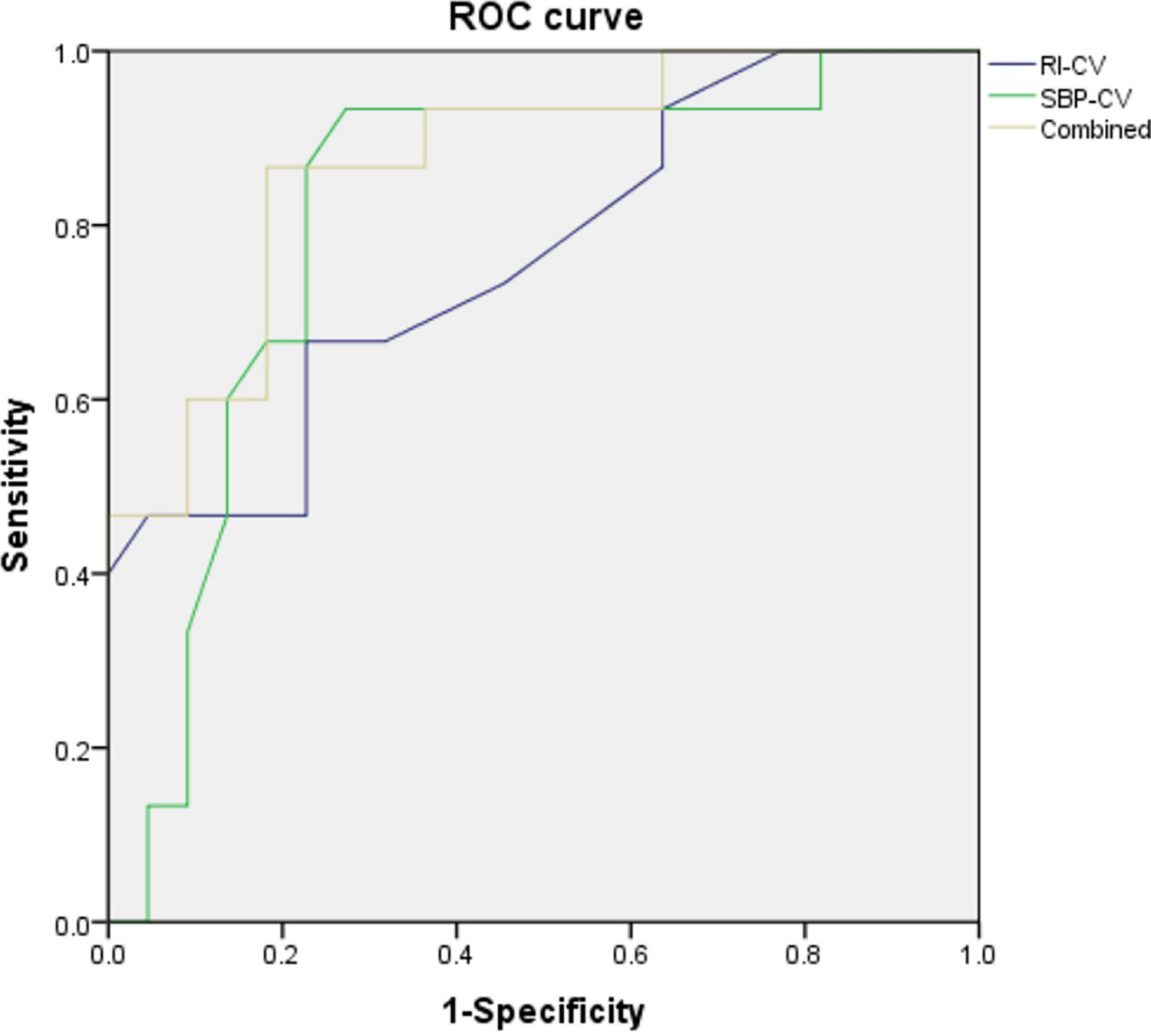
ROC curve analysis of risk factors for cerebrovascular complications: The ROC curves compare the predictive accuracy of RI-CV, SBP-CV, and their combination for cerebrovascular complications in neonates undergoing ECMO treatment. The blue line represents RI-CV, the green line represents SBP-CV, and the yellow line represents their combined model.

Table 3 summarizes the ROC curve analysis results of risk factors for cerebrovascular complications. The AUC for ACA-RI-CV was 0.765 (95% CI: 0.608–0.923, p = 0.007). The AUC for ACA-SBP-CV was 0.815 (95% CI: 0.666–0.964, p = 0.001). Moreover, when ACA-RI-CV and ACA-SBP-CV were combined, the AUC reached 0.873 (95% CI: 0.758–0.988, p<0.001) (Fig 2).

**Table 3:** ROC curve analysis of risk factors for cerebrovascular complications.

	AUC	95%CI	P value
RI-CV	0.765	0.608–0.923	0.007
SBP-CV	0.815	0.666–0.964	0.001
Combined	0.873	0.758–0.988	<0.001

## Discussion

The relative stability of cerebral hemodynamics is particularly important for neonates undergoing ECMO treatment. Although neonates possess mechanisms for cerebral blood flow autoregulation, these functions are not fully developed, making them more susceptible to autoregulatory dysfunction due to other influencing factors [12]. Additionally, neonatal cerebral vessels, especially in premature infants, are very small and fragile, making them prone to injury during hemodynamic changes [13,14]. Using blood pressure alone to indirectly reflect cerebral hemodynamic abnormalities in ECMO patients may not be accurate. Compared to this, combining cerebral hemodynamic assessment via cranial ultrasound with continuous monitoring of blood pressure fluctuations may provide a more accurate prediction of cerebrovascular complications in neonates during ECMO treatment [15,16]. Therefore, this retrospective analysis investigates the predictive ability of cranial ultrasound cerebral hemodynamic parameters for cerebrovascular complications in neonates undergoing ECMO treatment. Our results indicate that the incidence of cerebrovascular complications in neonates during ECMO treatment reaches 40.5%, which is consistent with findings by Sarah A. Teele et al. [17]. Our data show that neonates with higher variability in cerebral blood flow velocity have a significantly higher risk of cerebrovascular complications during ECMO treatment compared to those with lower variability. We also found that blood pressure variability was associated with the occurrence of cerebrovascular complications. Further validation through ROC curve analysis revealed that the variability of RI and blood pressure variability have good predictive value for cerebrovascular complications in neonates during ECMO treatment.

We found that neonates with high variability in cerebral blood flow velocity parameters have a significantly higher risk of cerebrovascular complications during ECMO treatment. RI is particularly effective in reflecting cerebral hemodynamics because it takes into account both systolic and diastolic velocities, providing a more accurate measure of microvascular resistance [18]. Therefore, this study also aimed to investigate the relationship between RI variability and cerebrovascular complications. It is known that cerebral hemodynamic changes induced by ECMO treatment can impair cerebrovascular autoregulation and increase the risk of cerebrovascular complications [19,20]. The significant increase in RI variability among neonates with cerebrovascular complications during ECMO treatment suggests that cerebral hemodynamics undergo significant fluctuations during this period, thereby increasing the risk of cerebrovascular complications. This finding highlights the necessity of routine cerebral hemodynamic monitoring during ECMO treatment and the potential value of such monitoring in preventing and managing related cerebrovascular complications.

In this study, we also explored the association between blood pressure variability and cerebrovascular complications in neonates receiving ECMO treatment. Blood pressure variability, or the degree of fluctuation in blood pressure over time, reflects the hemodynamic state of the body. Previous studies have shown that higher blood pressure variability was associated with the occurrence of cerebrovascular complications, and its predictive value for cerebrovascular complications in ECMO patients needs further analysis [21]. Our results indicate that neonates who develop cerebrovascular complications during ECMO treatment have higher blood pressure variability compared to those without complications. This suggests that neonates with greater blood pressure fluctuations during ECMO treatment have a higher risk of cerebrovascular complications. This finding was similar to results in adult populations, where such fluctuations may exceed the physiological regulatory range of cerebrovascular autoregulation, leading to cerebrovascular complications [22,23]. Therefore, monitoring blood pressure variability has certain value in the early identification and prevention of cerebrovascular complications in neonates undergoing ECMO treatment.

While it was clear that neonates on ECMO treatment with greater peripheral blood pressure fluctuations faced higher risks of cerebrovascular complications, it was still unclear whether peripheral blood pressure fluctuations could indirectly reflect the hemodynamic disturbances caused by cerebrovascular autoregulatory dysfunction in neonates. Hence, further analysis was required to assess the predictive ability of combined cerebral hemodynamic fluctuations and peripheral blood pressure fluctuations for cerebrovascular complications in neonates during ECMO treatment.

This study also investigated the predictive ability of combined RI variability and SBP variability for cerebrovascular complications in neonates during ECMO treatment. The results indicate that combined RI variability and SBP variability have predictive value for cerebrovascular complications in neonates during ECMO treatment. The AUC for combined ACA-RI-CV and ACA-SBP-CV in predicting cerebrovascular complications reached 0.873. Therefore, early high ACA-RI-CV and ACA-SBP-CV can partially reflect the state of cerebral blood flow and cerebrovascular autoregulatory dysfunction, providing some reference value for predicting cerebrovascular complications in neonates undergoing ECMO treatment.

## Limitations

Our study has several limitations. Firstly, this was a retrospective observational cohort study with a limited sample size, which restricts the generalizability of the results to some extent. Future studies with more rigorous prospective designs are needed to further explore these findings. Secondly, there may be inter-observer variability in the measurement of cranial ultrasound data. Although all ultrasound measurements in this study were performed by experienced cranial ultrasound specialists, individual differences cannot be entirely eliminated. Lastly, our study did not account for some potential confounding factors, such as variations in ECMO settings intracranial pressure, coagulation function, partial pressure of carbon dioxide, and arrhythmias. While all neonates in this study had their coagulation function and internal environment adjusted according to the same standards during ECMO treatment, it is still not possible to completely eliminate the influence of these confounding factors. Future large-scale prospective studies will be required to investigate these issues more thoroughly and improve the accuracy of the findings.

## Conclusion

High ACA-RI-CV and ACA-SBP-CV were associated with the occurrence of cerebrovascular complications during ECMO treatment in neonates. The combined detection of ACA-RI-CV and ACA-SBP-CV had a predictive role in the early identification of cerebrovascular complications in neonatal ECMO patients.
